# Courtship with two spoons—Anatomy and presumed function of the bizarre antennae of *Cardiocondyla zoserka* ant males

**DOI:** 10.1002/ece3.7615

**Published:** 2021-05-03

**Authors:** Jürgen Heinze, Jella Marschall, Birgit Lautenschläger, Bernhard Seifert, Nana Gratiashvili, Erhard Strohm

**Affiliations:** ^1^ LS Zoology/Evolutionary Biology Universität Regensburg Regensburg Germany; ^2^ Senckenberg Museum für Naturkunde Görlitz Germany; ^3^ Ilia State University Tbilisi Georgia

**Keywords:** antennal glands, Formicidae, Hymenoptera, mating behavior

## Abstract

Mating in ants often occurs on the wing during nuptial flights or on the ground when scattered female sexuals attract males by pheromones. In both scenarios, there is little opportunity for males to engage in prolonged aggressive competition or elaborate courtship displays. Male morphology is therefore adapted to locating female sexuals and mating, and it lacks specific weapons or other traits associated with courtship. In contrast, sexuals of the ant genus *Cardiocondyla* typically mate in their natal nests. As a consequence, in many species winged males have been replaced by wingless fighter or territorial males, which kill or expel rival males with their strong mandibles and show complex mating behavior. However, no wingless males are known from *Cardiocondyla zoserka* from West Africa, and instead, winged males have evolved a bizarre secondary sexual trait: uniquely shaped antennae with spoon‐like tips that show heavily sculptured ventral surfaces with numerous invaginations. We here report on the courtship behavior of *C. zoserka* males and describe antennal glands with class 3 gland cells, which presumably secrete a close range sex pheromone. Antennal glands have not yet been found in males of other ant species, including a close relative of *C. zoserka*, suggesting that in ants with intranidal mating sexual selection can rapidly lead to highly divergent adaptations and the evolution of novel structures.

## Graphical abstract

Winged males of the ant *Cardiocondyla zoserka* have evolved a bizarre secondary sexual trait: uniquely shaped antennae with a spoon‐like tips that show heavily sculptured ventral surfaces with numerous invaginations. We here report on the courtship behavior of *C. zoserka* males and describe antennal glands with class 3 gland cells, which presumably secrete a close range sex pheromone.

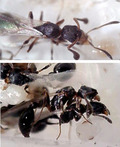

## 1 INTRODUCTION

Traits associated with courtship and mating can evolve rapidly and often result in striking differences in sexually selected traits among closely related species, for example, as in the displays of male bowerbirds, the coloration of male cichlid fish, and the song pulse rate in field crickets (reviewed by Broder et al., 2021; Svensson & Gosden, 2007; Zuk & Tinghitella, 2008). The morphology of ant males appears to be much more conservative and on a first glance differs little between congeneric species (e.g., Wheeler, 1910). The morphology and behavior of ant males are adapted to locating and approaching a female sexual and to mating. The typical mating syndromes of ants, male aggregation and female calling, do not give males much opportunity for elaborate courtship displays or male–male antagonism other than scramble competition (Boomsma et al., 2005; Hölldobler & Bartz, 1985). Ant males cannot easily obtain and defend a harem of queens and, like the males of other social Hymenoptera, start their sexual life with degenerated testes and a limited sperm supply sufficing for just one or a few copulations. As a consequence, weapons or costly ornaments as those found in many other animals are widely absent in ant males.

This is remarkably different in the myrmicine ant genus *Cardiocondyla*. Here, most matings occur in the natal nest, which has led to the evolution of nondispersing wingless (“ergatoid”) males. Wingless males either co‐occur with or have completely replaced the standard winged ant males (Kugler, 1983; Oettler et al., 2010). While winged *Cardiocondyla* males are similarly docile dispersers as other winged ant males and have not been observed to engage in male–male antagonism, wingless males of many species compete aggressively for mating chances within a nest (Heinze, 1999; Heinze et al., 1998; Kinomura & Yamauchi, 1987; Stuart et al., 1987). In several species, wingless males engage in fatal fighting, and successful males have been observed to remain as the only male in a colony for several weeks. As wingless *Cardiocondyla* males have re‐evolved life‐long spermatogenesis, they can regularly replenish their sperm supply and thus inseminate all female sexuals that eclose during this long period (Heinze & Hölldobler, 1993; Heinze et al., 1998; Yamauchi et al., 2006).

From the ancestral pattern of lethal combat, wingless *Cardiocondyla* males have evolved tactics that avoid the costs of fighting, for example, by focusing their attacks on freshly emerged, helpless males, by defending small territories in the natal nest, or even by completely tolerating other males (Heinze, 2017). This diversity of reproductive tactics is reflected in the morphology of the mandibles and genitals of wingless males, which show considerable differences among species (Schmidt & Heinze, 2018a, 2018b). Male courtship behavior shows similar variation, with males pummeling or hammering the heads of female sexuals often for minutes before the actual copulation ensues (Kinomura & Yamauchi, 1987; Mercier et al., 2007).

Winged males of *Cardiocondyla* are not pugnacious and also do not exhibit special morphological or behavioral traits adapted to competition or courtship, though to some extent they parallel wingless males in mandible shape and courtship behavior. Here we report on a striking exception in the West African species *C. zoserka*. Winged ants with strong mandibles and bizarre, spoon‐shaped antennae were originally described as female sexuals of a social parasite exploiting the colonies of another *Cardiocondyla* species (Bolton, 1982). However, a recent study revealed them to be the regular winged males of the presumed host species, which in contrast to all other studied *Cardiocondyla* appears to have no wingless males (Heinze, 2020). The uniquely modified antennae distinguish *C. zoserka* males from all other ants, while queens and workers of this species have “normal antennae” similar to those of males and females in other *Cardiocondyla* species. This suggested a role of the antennae of *C. zoserka* males in courtship and mating (Heinze, 2020).

We here portray the antennae in detail, give evidence for antennal glands—the first reported for ant males—and also describe male courtship behavior and male–male interactions in this species.

## 2 MATERIAL AND METHODS

### 2.1 Study species, collection, and maintenance of colonies

Two colonies of *C. zoserka* were excavated from their nests on sparsely overgrown sandbanks in the floodplain of Comoé River, Côte d’Ivoire, West Africa (8°46′2″N, 3°47′03″W, ca. 195 m elevation) in April 2019. *Cardiocondyla zoserka* appears to be much rarer than the morphologically very similar species *C. melana* and *C. venustula*, of which many more colonies were located in the same site. Colonies were housed for almost 1 year in incubators in the laboratory at University of Regensburg in plastic boxes with a regularly moistened plaster floor and a nest consisting of three parallel, 3 mm wide and 6 cm long slits in Plexiglas®, sandwiched between two microscope slides and covered by black foil. Colonies were kept at artificial 12 hr 28°C/12 hr 23°C day–night cycles and provided with honey and pieces of cockroaches twice per week (Heinze, 2020).

The two colonies survived until April 2020 and produced several dozens of winged males and winged female sexuals, and also a few workers. As in other tropical species of *Cardiocondyla*, queens appeared to be relatively short‐lived. From early 2020 on, colonies declined and the few surviving female sexuals, even though they had shed their wings, produced only males, suggesting that they had not been inseminated.

Voucher specimens from the two observation colonies are deposited in the ant collection of Senckenberg Museum of Natural History Görlitz, Germany.

### 2.2 Behavioral observations

Male behavior was studied in both colonies whenever males and female sexuals were available by ad libitum sampling (Altmann, 1974), that is, we focused on active males and tried to record as many activities as possible. In addition, we monitored male behavior over 3 × 12 hr day and night by snapshot‐recording using a Teslong MS100 Digital USB microscope camera (Teslong, Shenzhen, China). To facilitate copulation activity and prevent disturbance of courting pairs by workers, we also transferred males and female sexuals into small glass vials.

### 2.3 Histology

The antennae of three males were fixed in freshly prepared 2.5% paraformaldehyde/2.5% glutaraldehyde (Roth, Karlsruhe, Germany), buffered at pH 7.4 with 0.1 M cacodylate (Merck, Darmstadt, Germany) for 2 days at 11°C. They were postfixed in 1% osmium tetroxide (Science Services, Munich, Germany) in the same buffer, dehydrated in an ethanol series (Sigma‐Aldrich, Taufkirchen, Germany) and after a passage through acetone (Sigma‐Aldrich, Taufkirchen, Germany) embedded in EPON resin (Polysciences, Warrington, USA). Semi‐thin sections (1 µm) were prepared using an Autocut Reichert Microtome and a histology diamond knife, stained with toluidine blue, and examined by light microscopy (Zeiss‐Axiophot, Jena). Digital images were taken using Leica N Plan 40× and HCX Fluotar 100× oil immersion objectives (Leica Mikrosysteme, Wetzlar), and a Moticam 580 digital camera (Motic, Xiamen, China). Images were optimized for white balance and contrast (Adobe Photoshop elements 18).

Antennae from two additional males were fixed and dehydrated as above and air‐dried using chloroform (Sigma‐Aldrich, Taufkirchen, Germany), sputtered with gold/palladium (Polaron SC 515 SEM Coating System, Fisons Instruments, Glasgow), and examined with a JEOL JSM‐IT 100LV scanning electron microscope (JEOL, Tokyo, Japan) at 10 kV.

## 3 RESULTS

### 3.1 Behavioral observations

Despite of more than 16 hr direct observation and 36 hr by snapshot recording, we failed to observe complete copulations. Males did not approach female sexuals during the night, but were almost constantly trying to mate with winged female sexuals in the nest at daylight, particularly in the afternoon.

**FIGURE 1:**
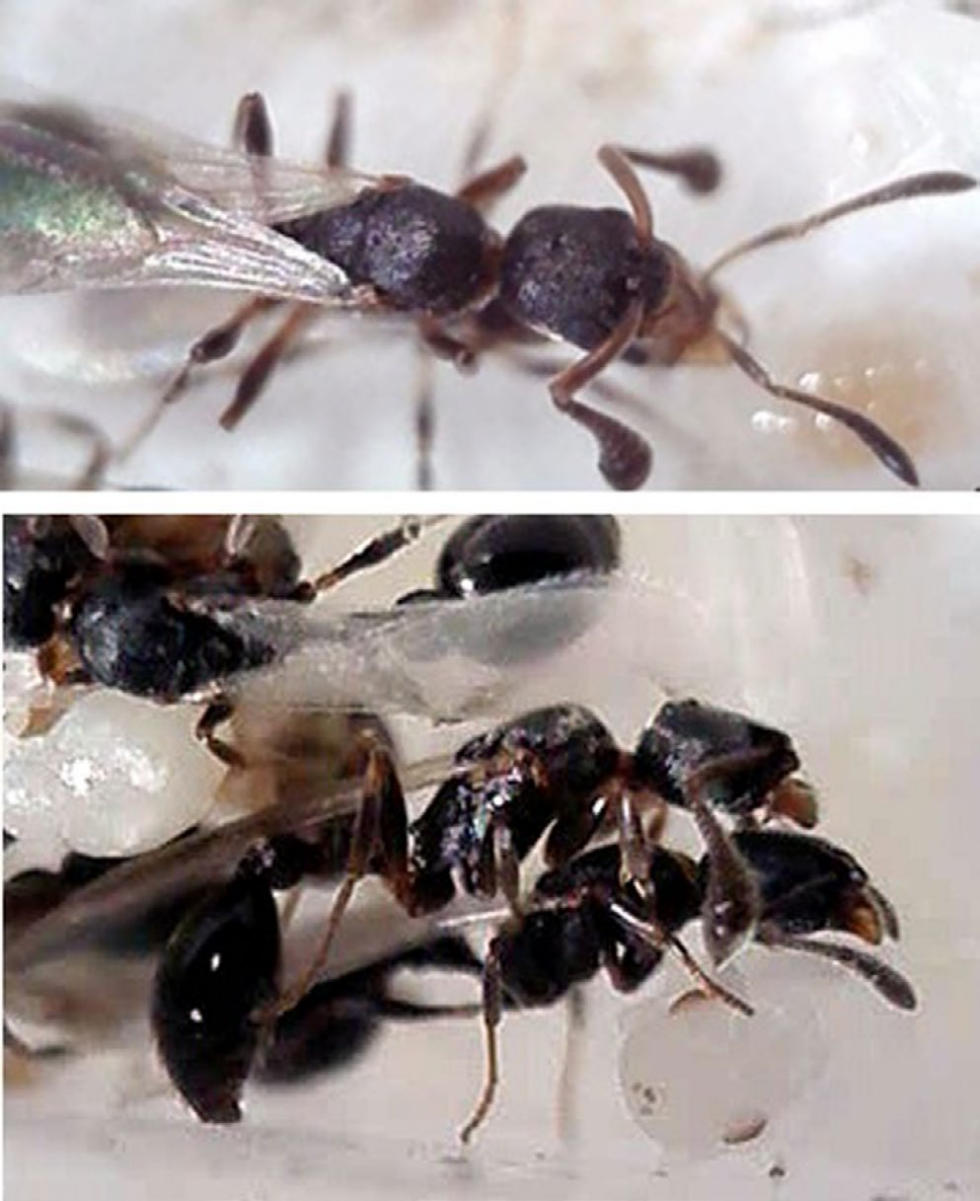
Male of the ant *Cardiocondyla zoserka* trying to copulate with a winged female sexual. In the upper figure, the position of the cup‐shaped antennal tip relative to the head of the female sexual can be seen; the lower figure shows that the male has extended its genital appendages, which are normally retracted and concealed

In a detailed analysis, consisting of 1–2‐hr bouts of continuous, focal sampling of male behavior spread over 3 weeks in December 2019, males antennated or courted female sexuals for 143 min of a total of 485 min observation time (29.5%; Table 1). When encountering a female sexual, males would climb onto its back, grasp its alitrunk with the legs, and stridulate, that is, rapidly vibrate their gasters. In at least one case, we observed that a male held the neck of the female sexual with its strong mandibles. Males clung to female sexuals for 4–45 min (median 13 min; 19 copulation attempts observed from beginning to end; Figure 1 and Video S1). They held their slowly moving or vibrating antennae close to the female sexual's head, with the concave side of the cup‐shaped tip directed backwards and toward the female sexual's head without touching it. Female sexuals initially appeared to tolerate the approach and remained immobile either with their antennae extended or appressed to the head. However, in one case the female sexual touched the cups of the male antennae with the tips of its own antennae for about 45 s. Males stayed attached to their partner even though the female sexuals clearly tried to shake them off by twisting their bodies or by rapidly moving through the nest. Even though males touched the abdominal tip with their extended genitals and in one case even ejaculated a droplet of sperm, female sexuals appeared to be reluctant to mate and instead pulled their abdomen under the thorax. Males were frequently seen approaching and antennating other males and apparently also tried to copulate with them (122 min, 25.2%). Overt antagonism between males was never observed.

**TABLE 1:** Percentage of time males of the ant *Cardiocondyla zoserka* engaged in different activities

Behavior	Total time (min)	Percentage of total observation time
Mating attempts with female sexuals	143	29.5%
Mounting other males	122	25.2%
Climbing onto workers	3	0.6%
Sitting without motion inside nest	27	5.6%
Sitting near food source outside nest	4	0.8%
Sitting near water source outside nest	2	0.4%
Moving in nest	61	12.6%
Selfgrooming	44	9.1%
Being groomed by workers	27.5	5.7%
Antennation with queen	13	2.7%
Antennation with other males	10.5	2.2%
Antennation with workers	9	1.9%
Being carried by workers	6	1.2%
Being pulled on antennae by workers	5	1.0%
Inspecting nest entrance	4	0.8%
Sitting with shivering antennae	4	0.8%

Note

As we were interested in documenting mating attempts, observations focused on periods during which males were active, that is, resting periods are greatly underrepresented.

As courtship generally occurred in the nest, workers and other males occasionally interfered, for example, by antennating or grooming the courting pair. Isolating female sexuals and males from the rest of the colony did not increase the success of courtship. Similarly, female sexuals were not more willing to mate when earlier contact to males had been prevented by isolating them as pupae together with workers or when they were confronted with a male from the other colony.

### 3.2 Morphology of the antenna of *Cardiocondyla zoserka* males

**FIGURE 2:**
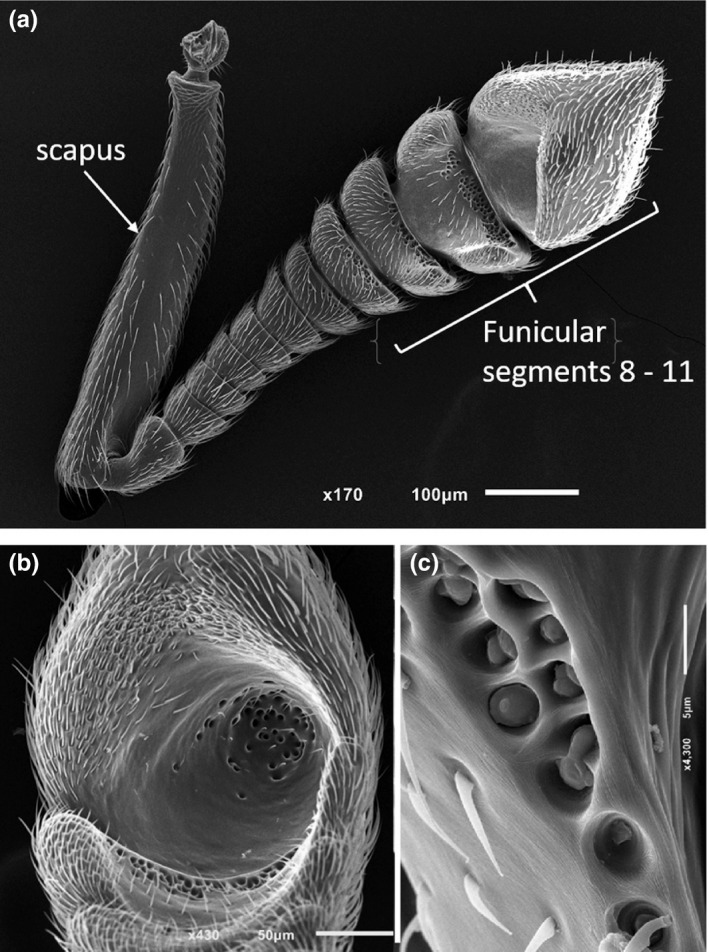
REM Images of an antenna of a male of the ant *Cardiocondyla zoserka*. (a) Whole antenna in ventral view, showing that the antennal segments are increasingly broadened toward the tip, with the distal antennomers showing numerous invaginations on their distal rim and the cup‐shaped, swollen apical segment (the image has previously been used to prepare figure 4 in Heinze, 2020). (b) Apical segment in ventral view with numerous sensilla and invaginations in the central pit. (c) Invaginations in the cuticle of the 9th funicular segment with emerging secretion

The antennae of *C. zoserka* males were already described in detail (though the males were then considered to be females) in the original description of the species (Bolton, 1982). As is typical for the antennae of many ant males, they are geniculate and consist of an elongate scapus and a funiculus, which in the case of *C. zoserka* consists of 11 segments (Figure 2a). In dorsal aspect, which is defined by viewing perpendicular on the swiveling plane of the 1st funicular segment (Seifert, 1988), the funicular segments 8–10 are extremely broad and the apical 11th funicular segment is “swollen‐conical” (Bolton, 1982). Funicular segments 6 and 7 are dorsoventrally flattened and while segment 8 is slightly transversely concave, segments 9 and 10 are very broad and strongly concave. The apical segment is invaginated and forms “a cup‐shaped hollow which extends deep into the segment” (Bolton, 1982). All funicular segments are covered by numerous sensilla. A closer inspection revealed heavily sculptured areas with numerous invaginations on the ventrodistal side of funicular segments 7–10 (Figure 2a), with their number increasing from the 7th (about 10 invaginations) to the 10th funicular segment (about 60 invaginations). Similar modifications of the cuticle could be seen on the ventral, proximal surface of the cup‐shaped apical segment (about 40 invaginations), which therefore superficially resembles a skimmer spoon (Figure 2a,b). Scanning electron microscopy of the invaginations suggested rests of secretions in some of them (Figure 2c) and also revealed a very finely striate microsculpture in the inner surface of the apical cup (see figure in Heinze, 2020).

### 3.3 Histology of the antenna

**FIGURE 3:**
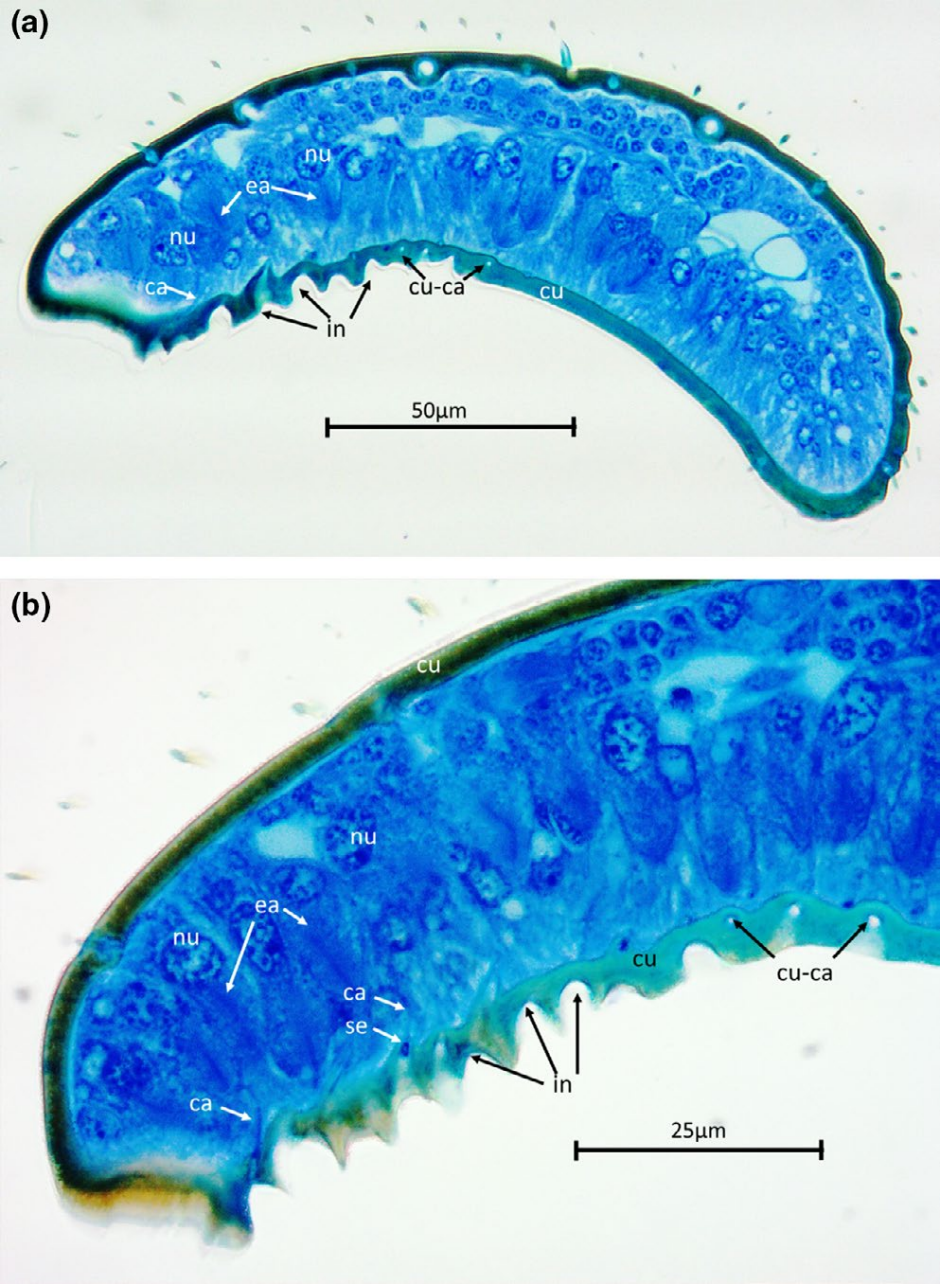
Longitudinal semi‐thin sections of the 9th funicular segment of a male *Cardiocondyla zoserka*, right side is apical, top is dorsal. The lumen is densely packed with differently sized cells. The cuticle at the proximal part on the ventral side is considerably sculptured by invaginations with openings of gland cells at their base. Secretions of the gland cells are visible in these openings. Abbreviations: ca, canal; cu, cuticle; cu‐ca, canal in cuticle; ea, end apparatus; in, invaginations; nu, nucleus of gland cell; se, secretion

Semi‐thin longitudinal sections of the 9th funicular segment of male antennae (Figure 3a,b) revealed a dark, that is, melanized dorsal cuticle and a lighter, that is, less melanized, ventral or inner cuticle. The proximal part of the ventral side of this segment was highly sculptured with numerous invaginations, some of which showed an intensively blue‐stained content. The segment was densely packed with cells. In the dorsal part, these were relatively small with small nuclei. In the ventral part, the cells were larger and elongated with large nuclei that showed numerous nucleoli. These elongated cells also exhibited structures that are most probably end apparatuses. This assumption is corroborated by the occurrence of canals that led from the region of the presumed end apparatuses through the cuticle to pores at the basis of the invaginations in the highly sculptured area (Figure 3a,b). Apparently, there was one canal and thus one gland cell per invagination. There were no indications of a gland reservoir, so the gland cells seemed to deliver their secretion directly to the openings without temporary storage.

## 4 DISCUSSION

The rare and inconspicuous African ant *Cardiocondyla zoserka* differs strikingly from its congeners by a number of extraordinary traits. First, it is the only species in the African *C. shuckardi* group (sensu Seifert, 2002) with winged males, and more generally, it appears to be the only known *Cardiocondyla* in which no wingless males are produced (Heinze, 2020). Second, the bizarrely shaped antennae of the male *C. zoserka* contain what appear to be the first antennal glands recorded for male ants, and behavioral observations indicate a role of these antenna in courtship and mating.

Our behavioral observations showed that males approached both related and, in experimentally staged encounters, alien female sexuals inside their natal nest. Similar to other males of *Cardiocondyla,* they climbed on the back of the female sexual and stridulated violently while clinging to their bodies with their legs and occasionally also with the mandibles. Interestingly, the antennae, though rapidly vibrating, were held relatively constantly at a distance from the female sexual's head. In one case, we could observe that the female sexual touched the cups of the male antennae with its own antennal tips.

The histological results suggest that in the ventroproximal part of the distal funicular segments there are several gland cells that show the typical structure of class 3 gland cells (Noirot & Quennedey, 1974): end apparatuses and canals. Each of these gland cells seems to deliver a secretion directly to the surface via pores at the basis of the invaginations in the associated sculptured area. The assumption that these are gland cells is corroborated by the fact that they have large nuclei with numerous large nucleoli, suggesting high metabolic activity. Since the number of gland cells is limited and there is no reservoir, only small amounts of secretion might be released. Since these glands occur only in males, a role in courtship seems plausible and the histological details suggest that males might deliver bursts of a close range sex pheromone at a certain time during courtship. The presumably very small amount of secretion will make it difficult to analyze its chemical composition.

Antennal glands occur in workers and queens of a few ant species. Class 3 glands have so far been found in the antennae of females of the myrmicine ants *Solenopsis* spp. and *Tetramorium bicarinatum* (Isidoro et al., 2000; Renthal et al., 2008), the ponerine *Dinoponera lucida* (Marques‐Silva et al., 2006), the formicines *Polyergus rufescens* and *Formica cunicularia* (Romani et al., 2006), and the army ant *Eciton burchelli* (Billen, 2000). The function of these glands in females is presently unknown. Isidoro et al. (2000) and Romani et al. (2006) explicitly mention that antennal glands were not detected in males of *Solenopsis invicta* and *Polyergus rufescens*, respectively, and other studies of male antennae also do not make references to glands, pores, or invaginations as we here describe for *C. zoserka* (Barsagade et al., 2013; Ghaninia et al., 2018; Nakanishi et al., 2009). Similarly, the examination of the antennae of an ergatoid male of *Cardiocondyla venustula* failed to reveal glandular openings (Heinze, 2020).

In contrast, antennal glands of class 3 appear to be quite widespread among males of other Hymenoptera, for example, bees, vespid wasps, and parasitoids, where they have been suggested to play an important function in mating (Isidoro et al., 1999; Klopfstein et al., 2010; Romani et al., 2003, 2005, 2008). In particular in parasitoid wasps, courtship often involves antennal stroking and coiling, and male parasitoids have evolved specifically modified antennae with internal glands that secrete contact pheromones (Klopfstein et al., 2010). For example, in the diapriid *Trichopria drosophilae*, during courtship the female touches with the tip of its antenna the 4th antennomere of the male antenna, whereby a sex pheromone from the male antenna appears to be transferred onto the female antenna (Romani et al., 2008). Similarly, the males of *Nomada* bees wind the flagella of their antennae around the female antennae and presumably apply a liquid pheromone secreted from the antennal gland (Schindler et al., 2018).

Males of other species of *Cardiocondyla* may use their normally shaped and presumably glandless antennae to stroke and pummel the head of the female sexual (Mercier et al., 2007). We hypothesize that an intense contact between female antennal tip and the male antenna might be part of the courtship in *C. zoserka*. At present, we do not know why all staged mating experiments in the laboratory failed. There might be several reasons: Our laboratory conditions might not have sufficiently matched those in the natural nests in extremely humid and warm soil, the food provided to the ants might have lacked certain compounds needed by males to produce glandular secretions, or female sexuals, though they typically remained inside the natal nest, might need some flight activity before they are ready to mate. Unfortunately, the presently unstable situation in West Africa and the impossibility to distinguish female castes of *C. zoserka* from related species without careful and detailed morphometry or genotyping will make it difficult to resume this study with new material of this amazing ant.

DNA sequences (Heinze, 2020) and the morphology of the female castes suggest *C. zoserka* to be closely related to the *C. shuckardi* species group (sensu Seifert, 2002). In *C. shuckardi,* C. *venustula*, and *C. melana,* all males are relatively large and ergatoid or “intermorphic,” that is, with the typical morphology of wingless males but short or rudimentary wings (Heinze et al., 2013). Considering that the estimated time of divergence between morphologically most divergent lineages of *Cardiocondyla,* such as *C. mauritanica* and *C. thoracica*, is about 20 million years (Ward et al., 2015), divergence time between *C. zoserka* and the *C. shuckardi* group should be much shorter. The evolution of antennae that are strikingly different in external architecture and inner tissue organization may thus have occurred rather rapidly, suggesting that intranidal mating in ants may occasionally lead to the fast evolution of highly diverging novel structures.

## CONFLICT OF INTEREST

None declared.

## AUTHOR CONTRIBUTIONS

**Jürgen Heinze:** Conceptualization (lead); formal analysis (lead); investigation (lead); writing–original draft (lead); writing–review and editing (lead). **Jella Marschall:** Investigation (supporting). **Birgit Lautenschläger:** Formal analysis (supporting); investigation (supporting). **Bernhard Seifert:** Formal analysis (supporting). **Nana Gratiashvili:** Investigation (supporting). **Erhard Strohm:** Formal analysis (equal); investigation (supporting).

## ETHICAL APPROVAL

Collecting of ants in Comoé Park was permitted by the director of the Office Ivorien des Parcs et Réserves (permit no. 221), the exportation of ants by a permit from the Ministère de l’ Enseignement Supèrieur et de la Recherche Scientifique. No other permits were required for the study.

## Supporting information

Video S1

## Data Availability

This research does not have additional data.
